# Occurrence, Sources, Phytotoxicity, and Prevention and Control System of Phthalate Esters in Cash Crops: A Comprehensive Review

**DOI:** 10.3390/plants15040549

**Published:** 2026-02-10

**Authors:** Shijie Ma, Shanjie Han, Jiankun Yuan, Cheng Pan, Qiaolei Cai, Mengxin Wang, Baoyu Han

**Affiliations:** 1 College of Life Sciences, China Jiliang University, Hangzhou 310018, China; s23090710033@cjlu.edu.cn (S.M.); s24090710067@cjlu.edu.cn (J.Y.); pancheng@cjlu.edu.cn (C.P.); s25090710002@cjlu.edu.cn (Q.C.); 2 College of Quality and Standardization, China Jiliang University, Hangzhou 310018, China; hanshanjie@cjlu.edu.cn

**Keywords:** phthalate esters, cash crops, occurrence, sources, response mechanism, integrated prevention and control system

## Abstract

Phthalate esters (PAEs) are emerging pollutants which are widely distributed in agricultural environments, and their impacts on crops have attracted considerable attention. PAEs on crops can disrupt their normal physiological metabolism, deteriorate the quality of agricultural products, and pose potential risks to human health through the food chain. Here, based on existing studies, we consolidate recent findings on the occurrence, sources, phytotoxicity, and control measures of PAEs in cash crops. Specifically, the pollution status of PAEs in cash crops was investigated. PAEs enter plants through water, soil, the atmosphere, and packaging materials via wastewater contamination, the degradation of plastic waste, and emissions from industrial processes. PAEs can induce oxidative stress in cash crops, disrupt photosynthetic pathways, and alter soil- and plant-associated microbial communities, leading to physiological and metabolic disorders that significantly reduce the yield and quality of cash crops. Consequently, recent studies have explored and developed more advanced mitigation strategies, such as enzymatic degradation, the use of microbial communities, and the development of new treatment materials and technologies. Overall, this review provides a comprehensive assessment of current research on PAEs in cash crops and offers insights into existing challenges and future prospects for ensuring the quality and safety of agricultural products.

## 1. Introduction

Phthalate esters (PAEs) are colorless, transparent, and oily liquids widely used in plastics, such as polyvinyl chloride materials, packaging materials, and children’s toys, owing to their excellent plasticizing properties [1]. They act as a “lubricant” for plastics, making hard plastics soft and durable [2]. They are bonded to plastic molecules through non-covalent interactions and can easily leach from products and enter the environment. This property has transformed PAEs from industrial aids to “invisible public enemies” in the environment. The widespread use of plasticizers has led to their prevalence in environmental media, creating a global environmental challenge [3]. PAEs are continuously released into the environment through various pathways such as industrial emissions, plastic degradation, and agricultural activities, resulting in the long-term accumulation of these pollutants in ecosystems [4]. In recent years, with the intensification of PAE pollution, crops have been found to absorb PAEs through root uptake, foliar adsorption, and atmospheric deposition, leading to significant bioaccumulation effects in agricultural products [5]. PAEs interfere with the normal physiological metabolism of crops, reduce the quality of agricultural products, and pose potential risks to human health through the food chain. Therefore, investigating the contamination characteristics, sources, phytotoxicity, prevention, and control measures of PAEs in crops is of great scientific importance for ensuring the quality and safety of agricultural products and managing environmental pollution.

Soil, water, and atmospheric deposition are key environmental media through which PAEs are transported to cash crops. PAEs can enter the soil environment through industrial wastewater discharge, the decomposition of plastic wastes, and the agricultural use of sludge [6]. Studies have shown that PAEs exhibit strong adsorption and persistence in soil, and they can be taken up through the root system and bioconcentrated in the edible parts of crops [5]. Water sources also serve as a major pathway for PAEs entering agricultural ecosystems. Industrial wastewater, domestic sewage, and surface runoff often contain high concentrations of PAEs, which can be transferred to soil and crops when such water is used for farm irrigation. The prevalence of effluent irrigation, particularly in water-scarce regions, further increases the risk of PAE contamination in crops. Atmospheric PAEs mainly originate from industrial processes, plastic incineration, and automobile exhaust emissions [7] and can enter agricultural environments through wet and dry deposition, adhering to crop surfaces or penetrating through the stomata. In addition, commonly used agricultural inputs such as plastic mulch films, pesticides, and fertilizers may also introduce PAEs into farmland [8]. The direct or indirect use of these agricultural inputs further increases the accumulation of PAEs in crops, and the limited capacity for crops to degrade these compounds further exacerbates the risk of contamination.

In recent years, owing to the chemical stability, strong adsorption capacity, and environmental persistence of PAEs, pollution control technologies that target them, particularly those based on agronomic control measures, have attracted widespread attention. Among these, biochar has emerged as an effective adsorbent for PAEs owing to its rich pore structure and stable physicochemical properties [9]. Distillation, mainly used in the pretreatment and refining of vegetable oils, is typically carried out through molecular or steam distillation processes [10]. Hydrolysis breaks down PAEs by cleaving chemical bonds, while microbial degradation involves the biological adsorption of PAEs by bacterial flora and enzyme-catalyzed reactions that initiate their degradation [11]. Although the above methods have achieved some success in PAE pollution control, they still have limitations such as complexity, low specificity, high costs, and limited overall efficiency. Therefore, there is an urgent need to explore and develop more advanced and effective treatment approaches. Recent studies highlight promising strategies, including enzymatic degradation [12], the use of microbial community consortia [13], genetically engineered microorganisms [14], microbial immobilization techniques [15], and the development of novel treatment materials and technologies that significantly enhance PAE degradation efficiency [16].

**Figure 1:**
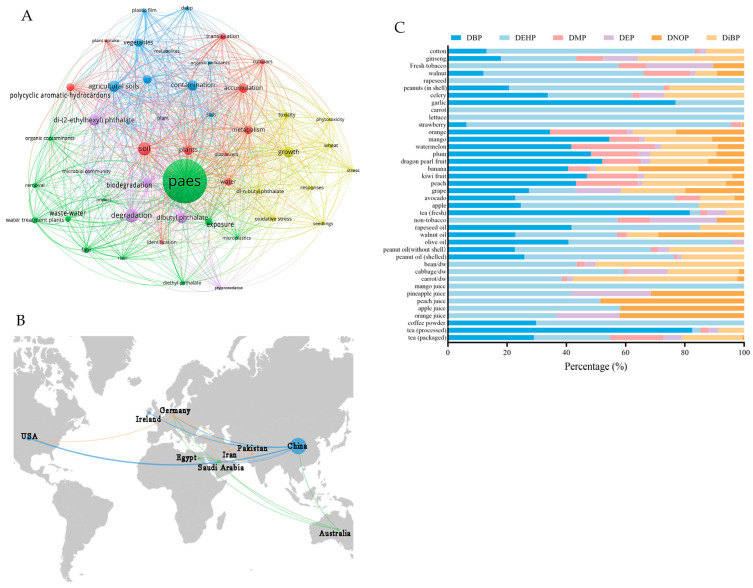
Studies on PAE pollution in plants published from 2008 to 2026 (January). (**A**) Network map of keyword co-occurrence for PAE research in plants. Node connections illustrate temporal associations, with line thickness and density reflecting keyword contribution strength. (**B**) The international collaboration network was visualized, where node size represents country-level research output, and link thickness indicates collaboration intensity. (**C**) The comparison of PAE profiles in cash crops. (Reference database: Web of Science; keywords: “PAEs,” “plants”; timeframe: 2008–2026; analysis software: VOSviewer.)

Currently, contamination of agricultural products by PAEs has attracted increasing attention [17]. Therefore, we used VOSviewer software (version 1.6.20) to construct a network map of keyword co-occurrence in plant-related PAE research [18]. The workflow, including detailed steps and parameter settings, is shown in the Supplementary Materials. As illustrated in Figure 1A, the keyword co-occurrence analysis identified “PAEs” as the largest node, while keywords such as “accumulation”, “growth”, “degradation”, and “agricultural soils” occur frequently in the literature published between 2008 and 2026 (updated in January 2026). These studies were conducted predominately in China, followed by the USA. Given China’s diverse and widely distributed crop systems, the country may face greater challenges related to PAE pollution in agricultural practices, which may have, in turn, driven more concentrated and in-depth research efforts; alternatively, Chinese researchers may show greater interest in this field. In addition, there is a relatively strong network of cooperation between countries. Therefore, existing studies mainly focused on the contamination characteristics, accumulation patterns, and remediation strategies of PAEs in crops, with a considerable proportion of research centered on Chinese agricultural systems. Wu et al. [19] conducted a systematic study on the contamination characteristics of seven PAEs and two phthalate monoesters (mPAEs) in crops in eastern China. Feng et al. [5] elucidated the uptake–transport–metabolism of PAEs in crops for the first time in a series of full-life-cycle processes. Their findings demonstrated that in phase I metabolism, PAEs are converted to various metabolites through hydroxylation, hydrolysis, and oxidation. In phase II, these metabolites are further combined with biomolecules such as amino acids or sugars. Some of these intermediate products might exhibit greater toxicity than the original compounds [20], raising concerns about their potential health and environmental impacts. Liang et al. [2] summarized the contamination characteristics, uptake, transformation, and toxic effects of PAEs on aquatic plants and highlighted that PAEs can lead to growth inhibition, structural damage, impaired photosynthesis, oxidative stress, and potential genotoxicity. Although important progress has been made in existing studies, research on species-specific responses is still insufficient. Before implementing advanced remediation strategies such as enzymatic degradation, microbial consortia, genetic engineering of microorganisms, microbial immobilization, and the development of novel treatment technologies and materials, a critical analysis of the contamination characteristics and sources of PAEs in crops is crucial. A systematic study of the distribution pattern, contamination sources, and prevention and control technologies of PAEs in crops is of great scientific significance and practical value. Such efforts are essential for ensuring the quality and safety of agricultural products and for promoting the sustainable development of agriculture.

Cash crops are an important part of agricultural production, with high economic value and strong market demand. Compared with food crops, cash crops usually have a longer industrial chain and higher value-added commodities and play a key role in increasing the income of farmers, driving structural adjustment in the agricultural sector, and promoting rural economic development. However, the intensive cultivation mode of cash crops may lead to an increased risk of exposure to PAEs. The aims of this paper are as follows: (1) to integrate and analyze the contamination levels of PAEs in cash crops, providing a comprehensive assessment of their current pollution status; (2) to examine the main sources of PAE contamination in cash crops; (3) to systematically describe the phytotoxicity of PAEs on cash crop physiology and quality; and (4) to review the effectiveness and prospects of both conventional and emerging technologies for PAE pollution prevention and control. Through this multi-dimensional analysis, the study seeks to enhance the scientific understanding of PAE contamination in cash crops and to provide a theoretical basis and technical support for risk assessment and quality and safety management of agricultural products.

## 2. Occurrence of PAEs in Various Cash Crops

**Figure 2:**
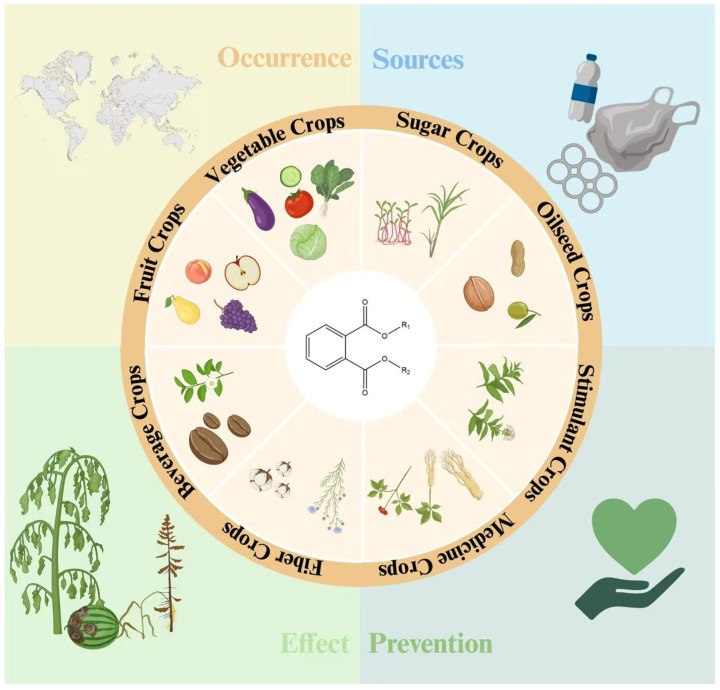
Cash crop classification and PAE contamination: occurrence, sources, phytotoxicity, and prevention and control strategies. Cash crops can be classified into the following types: beverage crops, fruit crops, vegetable crops, sugar crops, oilseed crops, stimulant crops, medicine crops, and fiber crops.

Cash crops are cultivated with the objective of market exchange and high economic value, which distinguishes them from staple food crops grown for local subsistence. They include a wide range of categories such as beverage crops, fruit crops, vegetable crops, sugar crops, oilseed crops, stimulant crops, medicinal crops, and fiber crops (Figure 2). In the present study, PAE data were collected from published research on cash crops and their products (Table 1 and Figure 1C). The analysis revealed that PAEs were detected in all examined cash crops and their products, indicating their widespread prevalence within the agricultural production system. Although the diversity of crop products and variations in extraction and analytical methods resulted in substantial differences in PAE composition and concentrations, the compositional characteristics of PAE contaminants in cash crops exhibited certain common patterns. Specifically, di-(2-ethylhexyl) phthalate (DEHP) and dibutyl phthalate (DBP) showed a pronounced dominance in cash crops, as they were consistently detected at the highest concentrations and accounted for the largest proportion of contaminants in both unprocessed primary agricultural products and processed products. These were followed by di-n-octyl phthalate (DNOP) and di-isobutyl phthalate (DiBP). This pattern is consistent with the compositional characteristics of PAEs in soil environments [21], likely because DBP and DEHP are readily adsorbed by soils and can persist for long periods, thereby increasing their likelihood of entering cropping systems. Consequently, PAEs can be taken up by cash crops through exogenous pathways. These routes of exposure have contributed to the widespread presence of PAEs in cash crops, particularly in highly industrialized regions.

### 2.1. Beverage Crops

Beverage crops, such as coffee and tea, hold an important position in international trade and play a strategic role in the socio-economic development of many developing countries.

As a globally consumed beverage, tea holds substantial cultural, economic, and health significance. Tea is the second most consumed beverage globally after water and has a large number of consumers throughout the world. In recent years, the contamination of tea with PAEs has attracted much attention. Wang et al. [48] detected the presence of PAEs in 15 tea plant varieties, with DBP being the main congener, followed by DiBP, although variation among varieties was not significant. One study reported a significant negative correlation between the PAE content in fresh tea leaves and air temperature, while a positive correlation was observed with the air quality index [48]. Tang et al. [23] analyzed the dynamic distribution of five PAEs at various stages of black tea processing and found that the baking process was the main stage of PAE emission, indicating that PAEs were significantly degraded during the processing of tea. The leaching rate of PAEs into tea infusion was found to be positively correlated with their solubility [49]. Ying [50] detected 16 types of PAEs in tea and investigated the migration pattern of DiBP and DEHP from plastic packaging to tea. Pan et al. [22] analyzed 204 packaged loose tea samples representing six major tea types and found that DBP and DEHP were the main contaminants. Among these, dark tea showed the highest levels of PAEs, which were significantly higher than those found in black and green teas [22]. Kashfi et al. [51] revealed the risk of microplastic and PAE release from tea bags, noting that German-brand tea bags released fewer microplastics than Persian-brand tea bags. DEHP and DiBP were the main contaminants, with their carcinogenicity warranting further attention. In addition, PAEs are widely present in the soils of tea plantations. Li et al. [24] detected PAEs in 114 soil samples from 38 tea plantations across 13 provinces in China. The level of soil contamination was influenced by geographic location, and intercropping tea with chestnut trees significantly reduced PAE concentrations in the soil. However, long-term ecological risks associated with DBP and DEHP remain and require continued monitoring [52].

Coffee, one of the major beverages globally alongside tea, is popular around the world. Studies have shown that PAEs are commonly detected in coffee [25], with concentrations varying considerably across different stages of production, including raw coffee, roasting, packaging, and brewing. PAEs have been found in brewed coffee samples in the range of 159–5305 μg/L [26]. Isci et al. [27] analyzed 40 coffee samples from different brands and packaging types, detecting the presence of benzyl butyl phthalate (BBP), DBP, DEHP, and diisononyl phthalate (DiNP). Their study estimated that individuals in the sampled population over the age of 15 were regularly exposed to PAEs through daily coffee consumption, raising concerns about cumulative exposure and its potential carcinogenic risks [27]. Velotto et al. [53] found no significant difference in the concentration of DBP and DEHP in coffee beans or powder across different packaging types. However, the coffee extraction method had an impact on PAE content: DEHP content in coffee extracted using a professional espresso machine was significantly higher than that in coffee extracted using a Moka pot and a home espresso machine, likely owing to leaching from machine components.

### 2.2. Fruit Crops

Fruits hold high economic value owing to their rich nutritional content, unique flavors, and wide range of applications. In recent years, research on PAE contamination in fruit crops has mainly focused on the fruits themselves. Zhang et al. [54] investigated various parts of bagged apples and found that the apple skin was the main site of PAE accumulation. Zhu et al. [39] detected six PAEs in 11 fruit samples, including kiwifruit, banana, dragon fruit, and watermelon, with DBP as the main contaminant. Meanwhile, Hou et al. [40] reported that PAEs penetrated the peel and accumulated in the pulp of apples and avocados, with concentrations reaching up to 0.188 mg/kg. In grapes from Turpan of China, eight PAEs were detected, with DBP, DiBP, and DEHP accounting for 95.4% of the total PAEs [55]. A survey of fruits from Zhejiang province in China showed the highest PAE concentration in citrus fruits, reaching 138.93 μg/kg [56]. Isci [57] analyzed 48 fruit juice samples from various brands and types available in Turkey and found DiNP, diethyl phthalate (DEP), and DEHP to be the most prevalent plasticizers. The finding indicated negligible non-carcinogenic health risks associated with the consumption of fruit juices across all age groups. However, the mixed fruit juices from Pakistan exhibited the highest PAE concentration and a high carcinogenic risk factor of 4.5 × 10^−3^ [58]. Packaging materials, orchard soil conditions, and agronomic practices influence PAE content in fruits. Zhang [42] reported that PAEs in peach fruits mainly originated from packaging materials and soil migration. However, dimethyl phthalate (DMP) was not detected in packaging materials, suggesting that it likely originated from the soil. Zhang et al. [59] examined the soil–strawberry system and found PAE contamination in both strawberries and soils, with DEHP and DiBP as the dominant congeners. Li et al. [60] observed that the total concentration of PAEs in grape-growing soils was significantly higher in greenhouse systems than in open-field conditions and increased with planting years. These results further pointed out that PAE contamination in grapes from Xinjiang in China was mainly related to the use of agricultural films, and the pollution level varied significantly across production regions [41].

### 2.3. Vegetable Crops

Vegetables, an essential part of the daily human diet, are rich in vitamins, minerals, and dietary fiber and play a key role in maintaining human health. However, they are also susceptible to PAE contamination during cultivation. PAEs can enter vegetables mainly through soil–plant system transport, irrigation water transport, and the use of agricultural plastic films. Existing studies have reported a 100% detection rate of PAEs and their metabolites in vegetable samples [19], with a concentration range of 0.19–56.8 mg/kg (average 8.07 mg/kg). Among them, leafy vegetables showed a significant characteristic of PAE accumulation [59]. By contrast, a survey found the highest PAE concentrations in root vegetables, likely owing to differences in sampling sources and environmental conditions. The level of PAE contamination in crops is influenced by several factors, including the physicochemical properties of individual PAEs, crop growth stage, uptake mechanisms, local environmental conditions, cultivation methods, climate, etc. [5]. Chen et al. [8] reported DEHP levels in vegetables ranging from 0.95 to 8.09 mg/kg. Lin et al. [61] further found that DEHP was more prevalent in both root and tuber vegetables and in leafy vegetables, whereas DBP concentrations were higher in *Lycopersicon* vegetables. Lettuce exhibited a special absorption preference for DBP [62]. Compounds such as DBP and DEHP, which have relatively large molecular weights, greater water solubility, and higher octanol–water partition coefficients, were more readily absorbed and accumulated by vegetables during cultivation, thus posing a potential risk to human health through the food chain [61]. In contrast, short-chain compounds such as DEP and DMP tend to leave lower residue levels in plants, owing to their high water solubility and easy biodegradation. Health risk assessments have shown that the hazard indices for PAEs in vegetables remain below one, indicating that the health risks are within acceptable levels [59]. A survey from Tianjin in China revealed that vegetable and orchard soils showed a significantly higher concentration of PAEs compared to the paddy and cotton soils [63]. Systematic studies have shown that in the vegetable–soil system, sources of PAE contamination include wastewater irrigation, fertilizer application, pesticide residues, and greenhouse plastic films [8].

### 2.4. Sugar Crops

Sugar crops, particularly sugarcane and sugar beet, are a group of important cash crops known for their high sugar content, and they play a vital role in global agricultural trade and the food industry. With growing global demand for sugar, the cultivation of sugar crops has expanded considerably. The extensive use of agricultural plastic films and the frequent fertilizer application under modern agricultural practices have led to increased PAE pollution in sugar crops, which has gradually attracted widespread attention.

Currently, research on the characteristics of PAE pollution in sugar crops is relatively limited and mainly focuses on PAE levels in planting soils. Guan et al. [64] reported an average PAE concentration of 736.5 μg/kg in four land-use types (sugarcane soil, paddy field, vegetable field, and fruit garden) in the Leizhou Peninsula, with DBP and DEHP identified as the main pollutants. The highest PAE contamination was found in sugarcane fields and was attributed to the extensive use of agricultural films during the nursery stage. In addition, sugarcane is typically grown in nutrient-poor soils that require high amounts of fertilizers, which may also contribute to high PAE levels [64]. Miriyam et al. [65] reported that *Saccharum officinarum* waste could serve as an alternative precursor for producing activated carbon, effectively removing DMP, DEP, and DBP from aqueous solutions. Sugar beet, another important sugar crop, has a well-developed tuberous root system with a strong capacity for exudate secretion. This trait plays a significant role in regulating soil carbon and nitrogen cycles and key enzyme activity. Wei et al. [66] found PAE concentrations in sugar beet to be approximately 0.45 mg/kg, with DBP and DEHP as the main contaminants. Intercropping with other plants reduced PAE content in sugar beet and enhanced the crop’s ability to remove PAEs from the soil. This effect was likely owing to the increased metabolic activity in the inter-root microbial community, which prompted the secretion of key enzymes such as catalase (CAT) by both roots and microorganisms, thus promoting PAE degradation.

### 2.5. Oilseed Crops

Oilseed crops are economically important crops whose seeds or germ tissues are rich in oils and fats, including soya beans, rapeseed, peanuts, and olives. Owing to their lipophilic nature, oilseed crops are more likely to absorb PAEs from the environment and further accumulate PAEs during processing. For example, peanut roots tend to accumulate PAEs and may even contribute to the removal of PAEs from contaminated soils [67].

Some studies have reported the presence of PAEs in 124 samples of 16 types of oilseeds from China, with DBP and DEHP being the most frequently identified. Total PAE concentrations ranged from 0.14 to 3.05 mg/kg, with *Cyperus esculentus* showing the highest contamination level [68]. No significant difference in PAE levels was observed between woody and herbaceous oilseeds. Fan et al. [67] analyzed 490 peanut samples from 17 provinces in China and found a 100% detection rate of PAEs, with an average concentration of 3.6 mg/kg. DiBP and DBP accounted for the largest proportion of PAEs detected. Among the tissues examined throughout the peanut growth cycle, PAE content was highest in the roots, with DBP and DEHP peaking during the pod and flowering stages. Notably, DEHP was detected in 100% of the peanut samples [69].

Research on PAE accumulation in raw materials of oilseed crops is relatively limited, whereas PAE content in oil products has received extensive attention. Significant amounts of PAEs have been detected in almost all the plant oil samples collected worldwide [70], and several studies have reported the prevalence of excessive PAEs in commercially available edible oils. Zou et al. [33] tested 33 batches of rapeseed oil and found DBP and DEHP in 93.9% of the samples. Shi et al. [71] reported that 38.2% of edible vegetable oils contained excessive levels of DBP. Jiang [34] found that walnut oil contained the highest DNOP and DEHP levels, up to 9.001 mg/kg, mainly originating from the oil extraction and walnut oil storage process. Liu et al. [72] also found that as plastic impurities in peanut kernels and rapeseeds increased, PAE concentrations in the resulting gross oil also increased. Oils extracted via the leaching method showed higher PAE contamination risks than those obtained through mechanical pressing. Deodorization in the refining process can effectively reduce the PAE content in edible oils. Nanni et al. [73] found that the high PAE contamination of commercially available Italian olive oils might be related to the absence of a refining process. Kang et al. [74] reported that deodorization effectively reduced the PAE levels in oil-tea camellia seed oil. In addition, molecular distillation technology showed superior effectiveness in removing PAEs from edible oils. Chen et al. [10] found that this technique was particularly effective in removing PAEs from sea buckthorn fruit oil at high concentrations, reducing DEHP residues to 0.668 mg/kg. However, this technique is prone to nutrient loss; therefore, it is critical to balance the efficiency of PAE removal with the retention of essential nutrients. PAEs have been detected not only in olive oil packaged in polyethylene terephthalate (PET) containers but also in glass, porcelain, and cardboard containers. This contamination is attributable to migration from the packaging and production process, including contact with industrial materials such as tubing and machinery components [75]. In addition to processing and storage processes, exogenous contamination in the growing environment is also an important source of PAE contamination. Soil contamination in oilseed-producing regions is common; for example, the average level of six PAE compounds in soils from major peanut-producing areas in Shandong province of China was reported to be 1.22 mg/kg [76]. Cecchi and Alfei [77] detected DEP and DBP in extra virgin olive oil species and hypothesized that contamination could originate from the olive. Some studies have reported that ground cover practices can significantly increase the contamination level of PAEs in peanut kernels, with DEHP and DBP being the predominant contaminants [76]. PAE contamination occurs throughout the entire edible oil production chain and requires multi-dimensional strategies such as raw material control, process optimization, and packaging material substitution for prevention and control. Future research will focus on developing novel removal technologies with high efficiency and low energy consumption, building a dynamic monitoring network of PAEs covering the whole industrial chain, and establishing strict limit standards and risk assessment systems.

### 2.6. Stimulant Crops

Stimulant crops, which are high-value-added cash crops, are widely cultivated and hold considerable economic importance in China. Existing studies have shown that PAEs can be transported to tobacco plants through the cultivation environment, highlighting the need to closely examine their contamination characteristics and associated ecological risks. Soleimani et al. [36] found that the PAE content of fruit-flavored tobacco was significantly higher than that in traditional tobacco and that DEHP accounted for the largest proportion of all tobacco samples. Fresh tobacco leaves exhibited high levels of PAEs, and improper disposal of tobacco waste posed a considerable environmental risk. Risk assessments showed that the non-carcinogenic risk indices for tobacco-related PAEs were all <1, and the carcinogenic risk indices of DEHP were <10^−6^, indicating that the risks were within acceptable levels. Zhang et al. [37] reported that the PAE concentration in tobacco leaves was 1.3 times that in the surrounding soil, which might be attributed to the physicochemical properties of PAEs. As high-molecular-weight, hydrophobic organic compounds, PAEs showed significant bio-enrichment effects in plants. PAEs were commonly found in the soils of tobacco fields, with DEHP and DBP as the main congeners, both showing a 100% detection rate. Importantly, the age of mulching is a key factor affecting the accumulation of PAEs. Studies have found that the PAE content in tobacco leaves is significantly positively correlated with the number of years of mulching, and the accumulation process follows a distinct pattern: a rapid accumulation in the first 8 years, followed by a plateau [36]. This pattern provides an important basis for the development of a scientific agricultural film management system.

### 2.7. Medicine Crops

The large-scale cultivation of medicinal crops can effectively alleviate the collection pressure of wild medicinal resources, support the relocation and conservation of endangered medicinal plant species, and contribute to the development of regional biodiversity. However, PAE contamination poses a potential threat to the sustainable development of medicine crops. Several studies in recent years have confirmed the widespread presence of PAEs in medicinal plants and their products. PAEs were detected in *Ganoderma lucidum* [78]. Analysis of ginseng revealed the presence of 19 PAEs, with DEHP detected in 100% of samples—at concentrations up to 0.17 mg/kg—followed by DBP and DiBP [46]. Guo et al. [79] found residues of DEHP and DBP in both honeysuckle water and Chinese medicine injections. Traceability analyses revealed that plastic greenhouses, years of cultivation, packaging materials, production environments, and storage conditions all affect the accumulation of PAEs in medicine crops [46]. Lu et al. [80] found that the accumulation of PAEs in ginseng leaves was significantly higher than that in the roots, and prolonged cultivation periods exacerbated the level of contamination.

### 2.8. Fiber Crops

Fiber crops, as important cash crops, hold a key position in the global agricultural trade. Sun et al. [81] found that a wide range of PAE congeners were prevalent in commercial sorghum samples, and PAE contamination exhibited a wide-area characteristic. A dose–effect study showed that DBP had a biphasic effect on cotton seed germination, and the activities of antioxidant enzymes showed a hormesis effect with increasing DBP concentrations and were significantly and positively correlated with malondialdehyde (MDA) content, confirming oxidative damage as the underlying mechanism. PAE contamination in cotton is mainly derived from cotton field soils and the use of land cover film. Xinjiang, the main cotton-producing area in China, experiences PAE accumulation in soils owing to multi-year planting, return of cotton stalks to the field, and extensive use of plastic mulch during cultivation [82]. One study confirmed that the PAE concentration of cotton field soil was positively correlated with the amount of residual agricultural film and negatively correlated with both the degree of film fragmentation and film thickness [83]. Although available risk assessment data suggest that the cumulative reproductive toxicity risk from long-term exposure to PAEs is within acceptable limits, current studies are limited to specific fiber crop types and regional samples. Moreover, they often overlook potential synergistic or antagonistic effects between contaminants and lack comprehensive whole-life exposure assessment models.

## 3. Pollution Sources of PAEs in Cash Crops

**Figure 3:**
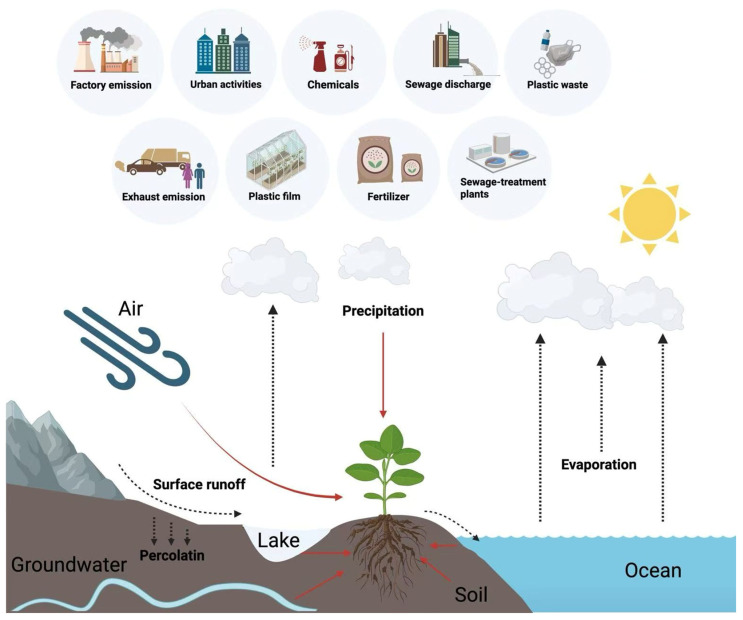
Pollution sources of PAEs in cash crops. Plants primarily absorb environmental PAEs through three major pathways: atmospheric deposition, soil uptake, and water absorption. PAEs may originate from multiple anthropogenic sources, including factory emissions, exhaust emissions, urban activities, fertilizer application, plastic film utilization, chemicals, direct sewage discharge, incomplete wastewater treatment from sewage treatment plants, and plastic waste.

The distribution of PAEs in the tissues of different cash crops exhibits significant variability, influenced by multiple factors such as crop species, growing environment, and tissue specificity. PAE pollution originates from diverse sources, with major exogenous input pathways including agricultural irrigation water, atmospheric deposition, migration from agricultural plastic films, the application of pesticides and fertilizers, etc. These pollutants enter the agricultural ecosystem through different pathways and subsequently bioaccumulate in cash crops (Figure 3). Although PAEs are widely present in media such as water, air, and soil, their concentrations vary across these media [84]. Therefore, a systematic analysis of the sources of PAE pollution can provide a theoretical foundation for developing targeted strategies for farmland pollution prevention and control.

### 3.1. Water

PAEs can enter the water environment through direct and indirect pathways. The direct pathway involves the discharge of industrial and agricultural wastewater containing PAEs and the leaching of plastic waste; the indirect pathway involves the emission of PAEs into the atmosphere and their subsequent migration to water bodies through wet and dry deposition or rainwater washing. In addition, freshwater algae and cyanobacteria can release mono-(2-ethylhexyl) phthalate (MEHP) or DBP into the aquatic environment [85].

The total concentration of water PAEs in China is within the range of 0.01–71,980 ng/L, with DBP being the most abundant congener in surface and drinking water, followed by DEHP and DiBP [84]. The similar composition of PAEs in surface and drinking water is likely owed to the fact that surface water is used as the drinking water source in most areas of China, and water treatment processes cannot effectively remove PAEs [86]. Bai et al. [87] systematically evaluated the removal efficiencies of conventional wastewater treatment processes for PAEs and showed that the removal effects were chemical-specific and process-dependent, and advanced oxidation technology showed good removal effects but was affected by the by-products. Liu et al. [88] used Taihu Lake as a research object and found that PAEs were significantly enriched in the lake water, suspended particulate matter, and plankton, with DBP and DEHP as the dominant congeners. They also found that the biological dilution effect significantly affected the distribution pattern of PAEs in plankton [88]. Zhang et al. [89] and Zhao et al. [90] investigated the pollution characteristics of PAEs in the Pearl River and Yitong River basins, respectively, and found DEHP and DBP as the main pollutants. These findings suggest a potential risk of PAE contamination in agricultural irrigation water through the water cycle. Similarly, Zhang et al. [91] found that DEHP, DiBP, and DBP dominated the PAE composition in the Yangtze River estuary and neighboring seas, and their concentrations were correlated with the salinity distribution, suggesting that river runoff inputs are the key drivers influencing the transport and diffusion of PAEs. In addition, the concentration of PAEs exhibits seasonal variation characteristics. A study conducted on Poyang Lake revealed that the concentrations of six PAEs in water ranged from 0.265 to 2.058 μg/L, with DBP being the main pollutant, followed by DEHP. There are significant seasonal differences in PAE concentrations in water. The concentration of PAEs in water during the dry period (November) was approximately twice the concentration during the wet period (July), which is likely related to the seasonal changes in rainfall, surface runoff, and water storage in Poyang Lake [92]. Zhao et al. [90] reported the highest PAE content in water samples from the Yitong River during the dry season. The researchers proposed two main contributing factors. First, increased emissions resulting from intensified human activity during the centralized heating period. Second, the low temperatures contributed to the brittleness of plastics and thus accelerated their release. However, seasonal patterns may differ in water bodies with different hydrological characteristics. The concentration of PAEs in the winter waters of Chaohu Lake was only half of that in summer. This phenomenon was likely due to summer rainfall depositing atmospheric PAEs and surface runoff-borne pollutants into the water, as well as resuspending sediment-bound PAEs.

In addition to their widespread environmental distribution, the significant ecological and human health risks posed by PAEs are well-documented and of substantial concern. Yin et al. [93] found that the co-existence of PAEs with perfluoroalkyl substances altered the bacterial community structure in drinking water systems and promoted the proliferation of pathogenic bacteria. Li et al. [94] revealed differences in the accumulation of six PAEs in leafy vegetables through hydroponic experiments and found that the enrichment capacity of the roots and the aboveground parts was closely correlated with the alkyl chain length and logKow values, which influence compound hydrophobicity and membrane permeability, thereby affecting bioaccumulation in plant tissues.

### 3.2. Atmospheric Deposition

PAEs in the atmosphere mainly exist in the gaseous or particulate phase [95,96,97]. Studies have shown that the concentration of PAEs in the particulate phase of the outdoor atmosphere in China is higher than the concentration in the gaseous state [98]. The main sources of PAEs in the atmosphere include release from traffic, industrial exhaust, plastic films used in agricultural activities, and fertilizer and pesticide application.

The total concentration of PAEs in the atmosphere in China was within the range of 0.004–33,851 ng/m^3^, with DEHP, DiBP, and DBP being the major contaminants [84]. Short-chain PAEs typically enter the atmosphere in vapor form through volatilization, while long-chain PAEs tend to adsorb onto fine aerosol particles or dust [97]. The spatial distribution of atmospheric PAEs varies across regions, influenced by meteorological conditions, local pollution levels, anthropogenic activities, sampling locations, and transport pathways of PAEs from emission sources to the atmosphere [99]. Kashyap and Agarwal [100] reported significant geographic differences in the concentration of PAEs in air and dust, with developing countries exhibiting higher pollution levels than developed countries. DEHP and DBP were identified as the main components. Moreover, factors such as ambient temperature, humidity, and construction materials significantly influenced the spatial distribution patterns of PAEs. When plastic films are released into the environment, PAEs undergo a complex redistribution process. Specifically, PAEs can either migrate from surface soil to the atmosphere through volatilization or return to the surface from the atmosphere through dry or wet deposition processes, thus forming a dynamic cycle at the soil–atmosphere interface until an interphase equilibrium state is reached [101]. The concentration of PAEs in the atmosphere shows a clear seasonal pattern of change. The increase in temperature accelerated the release of PAEs from the non-covalently bonded polymer matrix and the transfer from the particulate phase to the gas phase, leading to an increase in atmospheric PAE concentration. The concentration of PAEs in the atmosphere of Tianjin was reported to be significantly higher in summer than in winter (*p* < 0.05) [102]. However, Zhao et al. [97] found that the atmospheric PAE content in Wuhan was higher in winter than in summer and autumn, and this difference was possibly related to the effect of summer and autumn rainfall in Wuhan. In particular, hydrolysis and photodegradation contribute to the elimination of PAEs from atmospheric water-phase aerosols, but the efficiency of PAE degradation through these natural pathways is limited. In addition to temperature, relative humidity and wind speed also influences the distribution of PAEs in the atmosphere. Strong winds facilitate the dilution and dispersion of PAEs in the atmosphere, and high-humidity conditions can promote the aggregation of PAE-containing particles, leading to their eventual deposition onto the ground [103].

Although current atmospheric concentrations of PAEs are not high enough to cause immediate harm to ecosystems and human health, prolonged exposure to PAEs at low concentrations can result in their accumulation. Airborne PAEs can enter plants through gas or particle deposition, or both, penetrating the leaves and subsequently traveling through the phloem to reach plant bodies, including shoots, seeds, fruits, and roots [5]. Owing to the lipid-rich cuticle and the stomatal structure of plant leaves, foliar uptake is considered a major pathway for PAE accumulation in crops [104]. Studies have shown that PAE concentrations in mature leaves increase significantly with prolonged exposure time [49]. For example, *Brassica chinensis* and *Brassica camperstris* cultivated near plastic factories exhibited significantly high levels of DEHP in their leaves [105].

### 3.3. Soil Uptake

The main sources of PAE contamination in soil include urbanization and industrial activities, atmospheric deposition, and agricultural activities. Residual PAEs in soil adversely affect soil quality, which in turn impacts crop growth and quality, ultimately causing harm to animals and humans through the food chain. Current studies have shown that PAEs are prevalent in the soil–crop system with spatial and vertical variation. The average content of PAEs in agricultural soils ranged from a few μg/kg to tens of mg/kg, with the total content exceeding 100 mg/kg [84]; DEHP and DBP were the main pollutants, with some studies showing that high-molecular-weight DBP and DEHP were more likely to remain in the soil [106]. The exceedance rates of DEHP and DBP in soil and tobacco in the tobacco-growing region of eastern Guizhou in China were reported to be 55% and 100%, respectively [107].

Agricultural inputs and atmospheric deposition are the main sources of PAE contamination in agricultural soils [24]. Factors such as the material, color, thickness, and age of plastic products used in agricultural production influence soil PAE levels, and different types of agricultural plastic film materials significantly affect PAE release. The use of agricultural film has been confirmed to significantly increase soil PAE concentration [108], and the release of PAEs from agricultural residual film can last up to 60 years [109]. For example, the DBP and DEHP contents in soils of garlic production areas in Jiangsu province in China were significantly correlated with the residual agricultural film [110]. Cui et al. [76] reported that the total PAE content in soils of peanut production areas in the Shandong province of China ranged from 0.34 to 2.81 mg/kg, and film cover significantly increased the accumulation of PAEs. Wang et al. [110] found that the concentration of PAEs in soils of mulched farmland was 19% higher than that in non-mulched soils. PAEs are used as additives in fertilizers and pesticides. The application of organic fertilizers led to an increase in the accumulation of PAEs in soil and plants, with a doubling of PAEs in topsoil, suggesting that the use of organic fertilizers might have a significant effect on the dynamics and environmental behavior of PAEs in the soil [111]. Pot experiments by Cai et al. [112] showed that the total PAE content in *Vaccinium* plants treated with sludge-based organic fertilizers was significantly higher than that in the control group. Sewage sludge contained up to 32.41 mg/kg of PAEs when used as a soil conditioner [113]. Furthermore, analysis of 110 pesticide samples revealed that PAEs accounted for a range of 0.2–4% of organic solvents, with DEHP and DBP showing the highest detection rates among PAEs [114]. In addition, PAEs are also released into the atmosphere through exhaust emissions, pesticide spraying, and the degradation of plastic products. Once airborne, they can attach to particulate matter or precipitate onto soil, thereby increasing PAE concentrations. PAE contamination in soil shows spatial heterogeneity, with variations observed across different regions, even within the same area. Zeng et al. [115] reported the distribution characteristics of PAEs in agricultural soil in the suburbs of Guangzhou city in China for the first time and found that the concentration of PAEs was negatively correlated with the distance from the urban area, and the main pollutants were DiBP, DBP, and DEHP, which indicated that their accumulation was significantly correlated with the organic carbon content of the soil. Similarly, Liu et al. [116] showed that DEHP and DBP accounted for 76.8% of the total PAEs in soils across different functional zones in the Yellow River Delta, and oil extraction and chemical industry zones showed the highest level of contamination. However, the associated health risks were not thoroughly analyzed.

Pot experiments and model simulations showed that PAEs were most abundant in soil in the soil–plant–atmosphere system [117]. Soil moisture content also significantly affected the release and transport patterns of PAEs. An increase in soil water content was found to increase the transpiration rate of plants, leading to higher concentrations of PAEs in plant roots, stems, and fruits [101]. PAEs in soil can be absorbed passively or actively through the root system and transported upward through the soil–root–leaf pathway. In addition, the co-contamination of PAEs and microplastics in the soil of facility vegetable plots is intensified, and their transport behavior is influenced by the physicochemical properties of the soil [118].

### 3.4. Packaging Material

PAEs are commonly used as plasticizers and are widely used for packaging food materials. During the post-harvest handling of agricultural products, the problem of secondary contamination of PAEs has become increasingly prominent and cannot be overlooked. Recent studies have highlighted the growing risk of PAEs migrating from packaging materials into food, raising widespread concern. Beyond contamination from raw materials, PAEs and their migration rates are influenced by several factors, including the acidity of the food, storage temperature, and storage duration. The migration of PAEs in coffee and tea is related to their release from contact materials, preparation processes, and storage conditions [119]. Several studies have systematically examined the residue levels of PAEs in different types of packaging materials and found that the residue concentrations of DBP and DEHP in carton and cardboard packaging materials were within the ranges of 0.10–10,744 μg/kg and 0.52–61,013 μg/kg, respectively [120]. Some data showed that the detection rates of DEHP and DBP in food paper packaging materials reached 77.5% and 67.5%, respectively [121]. This high residue level may be attributed to the use of PAEs as additives in printing inks and coatings for food-contact materials [122]. Kashfi et al. [51] conducted an in-depth study on the migration behavior of PAEs from tea bags to tea infusions and found that the average concentration of PAEs in tea bags made in Persia and Germany was 2.87 mg/g and 2.37 mg/g, respectively, with DEHP and DiBP as their main contaminants, which posed a potential health risk. Pan et al. [22] detected PAEs in 204 packaged loose tea samples from six major tea types collected from 18 provinces in China and confirmed that packaging materials were an important source of PAE contamination in tea through source analysis. Migration of PAEs from plastic packaging materials to agricultural products is a major pathway of PAE accumulation, especially for cash crops with a higher oil content, which show a stronger PAE uptake capacity. Studies have shown that the migration behavior of PAEs from plastic packaging materials to oil-based food products is characterized by a rapid initial migration rate, followed by a continuous increase in migration over time. The total migration of PAEs is positively correlated with their initial concentration in the plastic packaging materials and the duration of contact with the oil phase [123]. The migration efficiency of PAEs into the oil matrix is influenced by their lipophilicity and molecular structure. For example, DEHP and DBP showed the greatest migration in edible oils, and their migration increased significantly with increasing temperature, contact time, and frequency of mechanical interaction [124]. Plastic is commonly used in pharmaceutical packaging, yet current quality standards for plastic drug packaging in China do not include PAE detection. Studies have reported that PAEs in moisture-proof sealing packaging materials migrated to *Ganoderma lucidum* spore powder and *Anoectochili*, leading to contamination [78,125]. Therefore, further attention should be given to the potential risk of PAEs migrating from packaging materials into medicinal plant products, and their migration pattern and safety impact should be systematically evaluated.

PAEs are transported to cash crops through environmental media such as soil, water, and the atmosphere. According to the aforementioned studies, there are systematic differences in the input pathways of PAEs for different categories of cash crops. The relative contribution of these pathways is highly context-dependent and is jointly influenced by crop physiological characteristics, cultivation patterns, regional environmental conditions, and agronomic practices. These exogenous inputs contribute to the accumulation of PAEs in cash crops, which may interfere with plant physiological metabolism and growth and potentially harm the human endocrine and reproductive system through the food chain. Hence, beyond identifying the sources of PAE contamination, it is crucial to further explore the phytotoxicity of PAEs on cash crops.

## 4. Phytotoxic Mechanism of PAEs in Cash Crops

**Figure 4:**
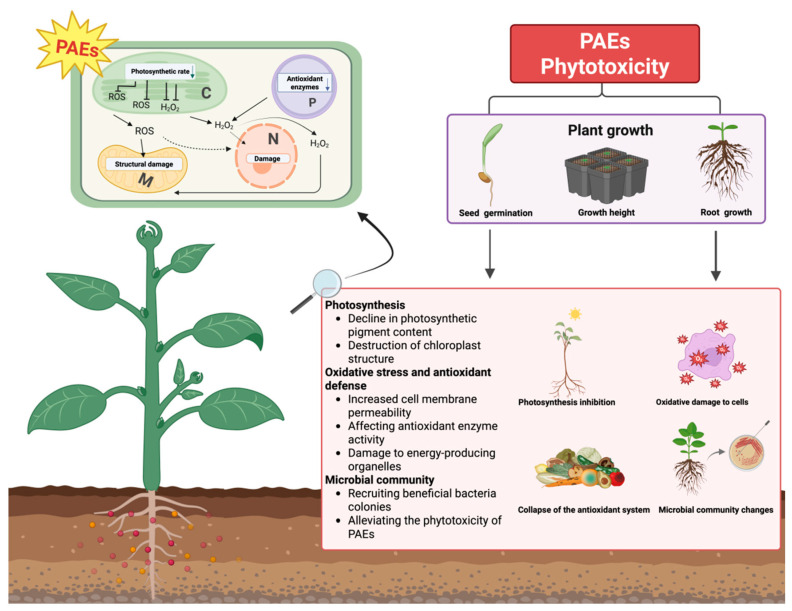
Effects of PAE phytotoxicity and morpho-physiological responses in cash crops. PAEs can adversely affect multiple physiological processes in plants, including growth regulation, photosynthetic efficiency, antioxidant system homeostasis, microbiome community structure, and reactive oxygen species (ROS)-mediated cellular damage. C: chloroplast; P: peroxisome; N: nucleus; M: mitochondrion.

As a typical environmental pollutant, PAE stress in cash crops and its toxic effects have become a major focus of research. Under stress from external pollutants, the nutritional quality and metabolic pathways of plants undergo significant alterations. Monitoring data indicate that the detection rate of PAEs in plants is approximately 100% [126]. When plants are exposed to PAE-contaminated air, PAEs may accumulate in the epidermal and waxy layers of leaves, stems, and fruits [126], particularly in leafy vegetables. Lower levels of PAEs have been reported in fruits than in leaves, probably because leaves have a larger surface area and are exposed to contamination for a longer period [127]. As a source of environmental stress in plants, PAEs can affect plant physiology, morphology, cellularity, and metabolism (Figure 4). Current studies on the phytotoxicity of PAEs on cash crops are mainly concerned with growth and development, photosynthesis, oxidative stress, and soil microbial communities. These effects vary depending on the concentration and types of PAEs.

### 4.1. Multilevel Impacts on Plant Ontogeny and Quality Determinants

Plant growth indices are one of the key biological parameters for assessing the toxicity of organic pollutants. Fan et al. [128] found that DBP significantly inhibited the growth of peanut plants from the seedling to the podding stage through a systematic study that applied multi-omics techniques. Another study reported that the growth height and fresh weight of *Brassica napus* grown in PAE-contaminated soil were significantly lower than those grown in uncontaminated soil [129]. Treatment of rape (*Brassica chinensis* L.) seeds with DBP and DEHP revealed that PAEs significantly inhibited seedling root length, shoot length, and fresh weight but had no significant effect on seed germination [130]. However, it was found that PAEs not only significantly inhibited the growth and development of cotton but also negatively affected its seed germination [131]. Herring and Bering [132] also found that DEP not only inhibited the root growth and shoot development of pea seedlings and spinach but also inhibited their seed germination. Under DBP stress, the germination rate of two cultivars of *Brassica rapa* var. *chinensis* was 20% lower than the control, and early growth was severely inhibited [133]. DMP can also inhibit the seed germination and growth of *Cucumis sativus* [134]. The root system plays a crucial role in plant growth and development and is sensitive to unfavorable growth conditions. PAEs not only impede crop growth but also significantly alter root morphology [135]. In addition, DBP adversely affected organic acids, vitamin C, and soluble proteins in cucumber fruits [136]; reduced vitamin C and capsaicin levels in capsicum [127]; and also reduced the nutritional quality of *Brassica napus*, including reduced soluble protein content and increased nitrate content [129]. In conclusion, the phytotoxicity of PAEs adversely impacts agricultural production by affecting plant germination and reducing yield and quality.

### 4.2. Impaired Photosynthetic Apparatus Functionality

The photosynthetic efficiency of plants is dependent on the light-trapping capacity of photosynthetic pigments and the subsequent photochemical reactions and carbon assimilation processes, which are core physiological characteristics that determine plant biomass accumulation and overall growth [137]. Ma et al. [138] found that total protein, soluble sugar, and free amino acid contents of lettuce leaves were positively correlated with the concentration of PAEs, while photosynthetic pigments contents were significantly inhibited by DBP. A study on the ecotoxicity of PAEs in cotton seedlings revealed that PAEs reduced the content of photosynthetic pigments and inhibited photosynthesis in cotton leaves, independent of growth effects [131]. Similarly, Xiao et al. [139] found that DEHP treatment significantly inhibited changes in photosynthetic pigments and chlorophyll fluorescence parameters in cucumber seedlings. Moreover, Zhang et al. [140] confirmed via cucumber seedling experiments that DEP caused more damage to chloroplast ultrastructure than DEHP. Xu et al. [141] found that the chlorophyll content in *Buckthorn multilocularis* under DEHP stress was reduced by 38.1%. The decrease in chlorophyll pigments is attributable to the production of free radicals, which disrupt the structure of chloroplasts, thereby inhibiting the normal photosynthetic capacity of the plant. Sun et al. [104] found that the co-presence of amino-functionalized polystyrene nanoplastics (PSNPs-NH2) and PAEs, through the down-regulation of transporter D1 protein expression, exhibited a stronger impairing effect on photosystem II efficiency, thus showing greater inhibition of plant growth. Yao et al. [130] found that DBP contamination significantly reduced the chlorophyll a, chlorophyll b, carotenoids, and soluble sugar contents of *Brassica napus* leaves and increased their proline content, thus adversely affecting the growth and quality of *Brassica napus*.

### 4.3. Induction of Oxidative Stress and Perturbation of Antioxidant Defense Systems

PAEs are mainly responsible for inducing oxidative stress in plants by generating reactive oxygen species (ROSs) and other intracellular or extracellular free radicals that affect plant metabolic activities, leading to membrane lipid peroxidation, increased cell membrane permeability, and electrolyte leakage. To mitigate the oxidative stress induced by PAEs, plants activate antioxidant defense systems including superoxide dismutase (SOD), CAT, and peroxidase (POD), as well as ascorbic acid and glutathione. These components work synergistically to neutralize ROS, maintain cellular homeostasis, and prevent oxidative damage [137]. DBP may damage energy-donating organelles such as chloroplasts and mitochondria, leading to the accumulation of ROS in the organism [130]. Excess ROS can further damage chloroplasts, leading to plant damage. Low concentrations of PAEs lead to an increase in the activity of antioxidant enzymes, indicating a “Hormesis”, where low-dose stress stimulates protective enzyme activity before becoming inhibitory at higher doses [142]. However, prolonged exposure results in the eventual dysfunction of antioxidant defenses, as the activities of enzymes such as SOD and POD first increase and then decrease, resulting in a collapse in stress resistance.

In plant physiological and ecological studies, plant resistance is enhanced by resistance substances and the regulation of enzyme activities from flowering to maturity [128]. DBP treatment of plants increased MDA content at lower concentrations but decreased it at higher concentrations [143], and the concentration-dependent decrease in MDA content may be due to an increase in the activity of the antioxidant defense system [144]. In addition, PAEs affect proline, hydrogen peroxide, free amino acids, and soluble sugar contents [137]. Proline is an osmoregulatory substance that maintains cellular osmotic balance and scavenges free radicals during oxidative stress, which can reduce cellular damage. Ma et al. [145] found an increase in proline, free amino acid, and soluble sugar contents when treating plant seeds with DBP and DEHP. DBP and DEHP also significantly increased the soluble sugar content of rape seedlings [146]. DBP induced an increase in proline and hydrogen peroxide contents in cucumber seedlings [147]. Plants under the phytotoxicity of PAEs may cope with it during germination by increasing resistance substances, a self-defense mechanism that occurs before the plant can synthesize sufficient protective enzymes [145].

In response to oxidative stress, plants activate enzymatic antioxidant defense systems that help maintain cellular homeostasis by detoxifying ROSs. DBP has been shown to increase MDA levels and antioxidant enzyme activities in *Brassica rapa* var. *chinensis* [133]. Similarly, Xiao et al. [139] found that DEHP significantly increased antioxidant enzyme activities in cucumber seedlings. One study confirmed that DBP and DEHP increased MDA content, SOD, ascorbate peroxidase (APX), and polyphenol oxidase (PPO) activities [146], and the affinity of PAEs for CAT increased with the growth of the alkyl chain in an experiment of rape contaminated with PAEs [130]. Lv et al. [131] found that PAEs interfered with the antioxidant system of cotton seedlings, caused lipid peroxidation damage, and also reduced the resilience and salt tolerance of cotton seedlings, which ultimately posed a serious challenge to the survival of cotton seedlings. In addition, Liu et al. [148] investigated the effects of PAEs on mung bean seed germination and antioxidant enzyme activities and found that the activities of SOD and CAT showed an increasing trend. However, an increase in the concentration of PAEs and the extension of the treatment time led to a decrease in SOD and CAT activities, showing a dose–effect relationship. Treatment with high concentrations of PAEs resulted in excessive accumulation of ROS, which exceeded the tolerance threshold of the plant, leading to the impairment of functional intracellular membranes and enzyme systems, ultimately inhibiting enzyme activities [149]. Yao et al. [130] found that DBP significantly down-regulated the antioxidant enzyme genes in *Brassica napus*, and the gene expression inversely correlated with the enzyme activities, implying that DBP might directly bind to the enzymes and interfered with their functions. Molecular docking analysis confirmed the stable binding ability of DBP to the active centers of SOD and POD.

### 4.4. Altered Plant-Associated Microbial Community Composition and Diversity

In addition to affecting plant physicochemical properties as described above, PAEs also affect plant-associated microbial communities, which are crucial for plant health and nutrition. These microbial communities play key roles in plant growth and development, nutrient uptake, stress resilience under abiotic stress, pollutant degradation, and pathogen suppression [17]. DBP has been shown to alter the alpha-diversity of phyllospheric microorganisms in field mustard at the five-leaf stage, significantly increasing the relative abundance of *Paracoccus* and *Rhodococcus* [150]. The structural and functional diversity of the rhizospheric microorganism community is an important factor influencing the phytotoxicity of PAEs. Ge et al. [133] reported that DBP contamination of *Brassica rapa* var. *chinensis* resulted in a decreasing trend in Chao1, Shannon, and Simpson indices of its root endophytic bacteria, while shoot endophytic bacteria showed an increasing trend in Chao1, Shannon, and Simpson indices. This finding indicates that DBP can alter endophytic and rhizosphere bacteria, which in turn affect the nutrient composition and ecosystem function of vegetables. Kong et al. [129] found that changes in the rhizosphere soil bacterial community of *Brassica napus* after DBP treatment were significantly correlated with the physicochemical properties of vegetables. Plants can recruit beneficial bacteria into the root interior and rhizosphere after PAE stress, and these specific bacteria can transform PAEs through direct degradation or co-metabolism, thus alleviating the physiological stress of PAEs on plants [5]. Studies have shown that endophytic flora can not only effectively degrade PAEs but also promote plant growth [151]. However, the microbial regulatory mechanisms of PAEs in plants are still poorly understood, and a more comprehensive exploration of the interactions among plants, PAEs, and their associated microbiomes is needed.

PAEs cause physiological and metabolic disorders and significant reductions in quality indicators by inducing oxidative stress and interfering with photosynthetic pathways in cash crops. Specifically, PAEs promote the excessive accumulation of ROSs after entering the plant body, which disrupts the dynamic balance of the intracellular oxidation–antioxidation system. This oxidative stress not only causes damage to cell structure, such as by inducing lipid peroxidation of the plasma membrane, protein denaturation, and nucleic acid degradation, but also interferes with photosynthesis through multiple mechanisms. These mechanisms include inhibition of chlorophyll biosynthesis, disruption of the electron transport chain of photosystem II, and reduced activity of photosynthetic enzymes. The cumulative effect of the oxidative stress is a decline in organic matter synthesis, which manifests as reduced germination rates, inhibited plant growth, and reduced cash crops quality. Remarkably, microbial communities play an important role in mitigating the phytotoxicity of PAEs. Physiological regulation mediated by specific functional microorganisms can effectively alleviate the metabolic disorders induced by PAEs in plants, thus maintaining plant growth performance and quality characteristics to some extent (Figure 4). Furthermore, under actual farmland conditions, PAEs often occur as part of combined pollution with heavy metals, pesticides, and microplastics, which substantially increase risk complexity. Microplastics can act as carriers for PAEs, facilitating their environmental transport and gradual release, while heavy metals and pesticides may exert additive or synergistic toxic effects with PAEs, collectively inducing oxidative stress or endocrine disruption. Environmental exposure levels directly influence the accumulation of active metabolites, such as phthalate monoesters, in plant tissues. These metabolites, for example, MEHP, exhibit higher toxicity than their parent PAEs [134] and can enter the human body directly through dietary intake. Consequently, monitoring only parent PAE residues in cash crops leads to a systematic underestimation of associated health risks. Future risk assessments should therefore incorporate the formation and contribution of plant-derived metabolites to achieve a more comprehensive evaluation of combined ecological and human health risks in agricultural environments.

## 5. Integrated Prevention and Control System

**Figure 5:**
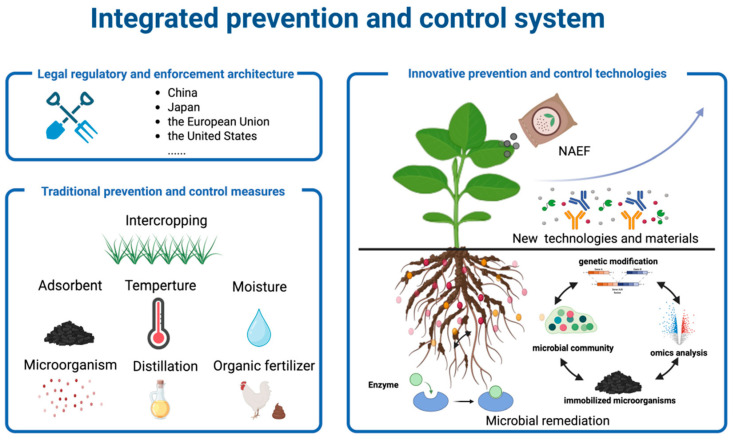
Integrated prevention and control system for PAE contamination in cash crops. Control measures primarily include regulations, traditional methods, and innovative control technologies.

PAEs not only inhibit cash crop growth and development, leading to lower crop yields and quality deterioration, but also pose multiple hazards to human health through the bioconcentration effect in the food chain, including but not limited to endocrine disruption and reproductive toxicity. Given the widespread contamination and serious implications of PAE pollution, establishing a systematic prevention and control system is essential in both environmental science and sustainable agriculture. From a strategic perspective, a comprehensive governance framework with three dimensions (regulatory measures, agronomic practices, and technological innovations) can be constructed (Figure 5). At the regulatory level, strict PAE limit standards should be formulated, particularly for food packaging materials. A comprehensive regulatory mechanism should be simultaneously established to strengthen the effectiveness and ensure proper compliance with regulations and standards. Traditional management technology is mainly based on agronomic control measures, while the new management technology is based on the research and development of new functional materials, the construction of efficient microbial flora, and the integration of multiple treatment technologies. This multi-level prevention and control system not only reduces the environmental risk of PAEs but also provides a theoretical basis and technical support for achieving synergistic management of agricultural product safety and ecological environmental protection.

### 5.1. Legal, Regulatory, and Enforcement Architecture

The potential environmental and health risks of PAEs, a class of widely used plasticizers, have triggered scientific concern and regulatory attention on a global scale. Owing to their ecotoxicity and health hazards, the international community has gradually established a targeted regulatory framework. Through scientific assessment and risk classification, differentiated restrictions and bans have been formulated, and comprehensive control measures have been implemented throughout the production chain, from production and import/export to market circulation, to reduce the circulation of high-risk products and protect ecosystem stability and public health. Currently, international regulation of PAEs mainly focuses on food-contact materials, aiming to reduce human health risks by limiting their migration into food. However, limit standards for PAEs in raw agricultural products, including cash crops, remain undefined in most national and international frameworks.

China has gradually established a PAE control system since 2003, with restrictions on the use of DEHP, DBP, and other PAEs in food packaging materials and by setting migration limits. China, the European Union (EU), Japan, and other countries and regions have successively improved the migration limits and testing method systems for PAEs in food-contact materials and products, toys, children’s products, and cosmetics. The U.S. Food and Drug Administration (FDA) controls PAEs through the Premarket Food Contact substance Notification system (FCN), which prohibits the use of specific PAEs, such as DEHP and DBP, in food-contact materials and sets strict migration limit standards for permitted PAEs. For example, the total PAE content in food-contact plastic products must not exceed 10% at room temperature. The EU, recognizing the endocrine-disrupting potential of PAEs, has identified maximum tolerable specific migration limits (SMLs) for PAEs and restricted the use of PAEs in plastic materials intended to come into contact with food (Directive 2002/72/EC, as amended). These regulations limit five PAEs: DBP, DEHP, BBP, DINP, and bis(8-methylnonyl) phthalate (DIDP) (Commission Directive 2007/19/EC, amending Directive 2002/72/EC) [26]. Notably, EU regulation No. 10/2011, governing the use of plastics in contact with food, was revised and came into effect on 31 August 2023. Under this regulation, the SML for DEHP is 0.6 mg/kg. Moreover, a combined SML of 0.6 mg/kg applies to the sum of DEHP and DiBP, while the total SML for DINP is set at 1.8 mg/kg [75].

Since the plasticizer scandal in Taiwan in 2011, China has introduced new national food safety standards and related regulations. In the same year, China mandated all local government supervisory bodies to conduct comprehensive inspections of food-related enterprises. They also established maximum residue levels of DEHP, DINP, and DBP in food and food additives, particularly requiring that the total amount of PAEs in flavors and fragrances used for food should not exceed 60 mg/kg. In 2019, China’s State Administration for Market Regulation (SAMR) issued the “Guidance Opinion of SAMR on the Risk Control and Prevention of Contamination in Foods”. This document formally clarifies the maximum residue levels of PAEs in oils and fats and alcoholic foods. The contents of DEHP and DBP in liquor and other distilled spirits should not be higher than 5 mg/kg and 1 mg/kg, respectively. The document also outlines limits for DEHP, DINP, and DBP in oils and fats and alcoholic foods (except liquor and other distilled spirits). It serves as a valid administrative norm and provides an important basis for the regulation of PAEs residues in food in the absence of uniform national standards. Although the Food Safety Law of the People’s Republic of China does not contain direct provisions on plasticizers, it indirectly controls them by incorporating them into the food safety standard system, reflecting the holistic framework of food safety regulation in China. In the future, with a deeper understanding of the environmental behavior and health risks of PAEs, the establishment of a standard system for the control and regulation of PAEs covering the whole chain of agricultural products will become an important development direction.

### 5.2. Traditional Prevention and Control Measures

Agronomic control measures offer several advantages, including environmental sustainability, low economic cost, in situ remediation capability, and absence of secondary pollution. These methods can simultaneously achieve agricultural productivity and soil remediation, making them increasingly recognized as ideal strategies for addressing PAEs contamination in crop soils [152]. Currently, key agronomic approaches for reducing PAEs levels include water regulation, temperature regulation, addition of soil amendments, and bioremediation.

Water regulation can affect the chemical degradation of PAEs in soil by directly influencing soil redox potential, pH, and physicochemical properties. Hydrolysis reactions are usually influenced by a combination of factors; although they can initially degrade PAEs, the hydrolysis efficiency is not high, and the hydrolysis cycle is long under natural conditions. In addition, water regulation may alter the abatement of PAEs in soil by affecting soil microorganisms. One study found that 60% water-holding capacity in soil was an appropriate moisture for DEHP dissipation [153]. Temperature is an important factor affecting the microbial transformation of PAEs, and increasing the temperature can stimulate the growth activity of microorganisms and enhance the solubility of PAEs in the soil, thereby increasing the rate of PAE degradation and transformation [152]. Biomass charcoal has a strong adsorption capacity for PAEs, and its mechanisms are mainly pore filling, hydrophobicity, hydrogen bonding, and π–π electron donor–acceptor interaction (EDA) [135]. Biomass charcoal has been shown to significantly inhibit the migration of PAEs to *Brassica napus*, thereby reducing their bioavailability [154]. Rao et al. [155] incorporated peanut shell biomass charcoal into DMP-contaminated soil and found that it could improve the adsorption performance of soil for DMP. Modified biomass charcoal can inhibit the phytotoxicity of PAEs in wheat seeds [156]. In addition, chitosan, activated carbon, and polymer resins are also important adsorbents for PAEs [21]. Chitosan is biodegradable in the environment. Chen and Chung [157] used chitosan for the adsorption of six PAEs and found that the adsorption increased as with increasing molecular hydrophobicity of the PAEs. In addition, exogenous addition of organic fertilizers had a positive effect on the reduction in PAEs in soil. For example, poultry manure ameliorated the negative effects of PAEs on microbial populations and soil enzyme activity, thus reducing the ecological risk of PAEs [158]. Some studies have shown that co-application of adsorbents as fertilizer carriers with organic manure can effectively promote the degradation of PAEs in soil [159], but the chemical and microbiological mechanisms regarding the promotion are yet to be thoroughly investigated.

For cash crop-derived products, molecular distillation has proven effective in reducing the DBP content in walnut oil from 1.53 mg/kg to 0.12 mg/kg and the DEHP content from 4.83 mg/kg to 0.98 mg/kg [160]. Compared with steam distillation, molecular distillation can remove PAEs from vegetable fats and oils more efficiently at lower temperatures and in shorter processing times, particularly for DBP and DEHP. Han et al. also found that the supramolecular solvent system could efficiently extract PAEs from edible oils with good sample clean-up [161].

In addition to the physicochemical remediation methods discussed above, bioremediation technology has become an important way to manage PAE pollution owing to its environmental friendliness and cost-effectiveness. Bioremediation mainly includes phytoremediation and microbial remediation. Phytoremediation involves the use of plants capable of accumulating or degrading pollutants to remove contaminants from the substrate, thereby reducing the risk of PAE transfer from soil to crops. Plant species such as alfalfa [162], legume-grass [163], *Elsholtzia splendens* [164], *Sedum plumbizincicola* [164], *Zizania latifolia* [165], *Lactuca sativa* L. var. *longifolia* [166], *Phragmites australis*, and *Typha orientalis* [167] showed potential for remediation of soils and wetlands contaminated by PAEs. Wei et al. [66] reported that sugar beet–alfalfa intercropping was more effective than monocropping, removing up to 66.48% of PAEs. Li et al. [52] found that compared with monocropping tea plantations, intercropping chestnut–tea plants significantly reduced the PAE content of tea plantation soils, which further verified that the driving factors of chestnut intercropping, temperature, and agrochemicals had a strong direct effect on the changes in PAEs in tea plantation soils. In addition to the different absorption and accumulation capacities of PAEs by plants of different species, different varieties within the same plant species also display significant differences in PAE uptake and accumulation. Screening and cultivating low-accumulation cash crop varieties is a highly effective strategy for promoting clean agriculture and optimizing agronomic regulation for PAE remediation. Some studies have found that root zone regulation and longitudinal translocation lead to inter-varietal differences in PAE accumulation in vegetables [168]. Zeng et al. [169] found that root secretions from two genotypes of oilseed rape had stronger desorption effects on DEHP in the soil. The two cultivars (Huaguan and Lvbao) of *Brassica parachinensis* significantly reduced the content of DEHP in the rhizosphere soil, mainly attributed to significant changes in the ecological network of bacterial communities, with *Chitinophagaceae* and *Nocardioidaceae* playing an important role in the biodegradation of PAEs [170]. Tang et al. [171] found that 2 out of 12 lettuce cultivars exhibited relatively low DEHP accumulation, mainly because their rhizosphere bacterial communities enhanced their adaptive capacity by enriching anti-pollution taxa, increasing extracellular polymer content and biofilm formation, and building complex ecological networks. Although some phytoremediation studies have been conducted on PAEs-contaminated soils, documented candidate plant species for practical remain scarce, and the efficient remediation mechanism is still unclear and requires further in-depth research.

Currently, microbial remediation has become an effective method for removing PAEs from soil owing to its high efficiency, low cost, low energy consumption, and absence of secondary pollution. Microbial remediation is the use of the natural ability of microorganisms to degrade or convert PAEs into less harmful substances [172]. Since the first report of microbial decomposition and metabolism of PAEs in 1975, many microorganisms capable of degrading PAEs have been isolated and characterized from a variety of samples. To date, over 80 strains of PAE-degrading bacteria from more than 30 genera have been reported and studied [21], including *Bacillus* [173], *Gordonia* [17,174], *Rhodococcus* [175], *Pseudomonas* [176], *Actinobacteria* [177], and *Streptomyces* [178]. A DBP-degrading bacterial strain, *Priestia megaterium* P-7, was isolated from the soil of a cotton field under long-term plastic mulching and found to have good degradation efficiency, substrate versatility, and environmental tolerance [179]. Wang et al. [180] isolated a DMP-degrading bacterium (DNM-S1) from a vegetable greenhouse, which exhibited significant degradation properties, and its secondary metabolites contained natural carotenoids that counteracted the disruption of cell membrane permeability by PAEs. Yuan et al. [181] isolated a DBP-degrading strain, JR20, from garlic chive, and found that the colonization of this strain significantly increased the rate of DBP degradation in the roots, stems, and leaves of leafy vegetables. In addition to bacteria, fungi and algae can also degrade PAEs. Wang et al. [182] found that six species of microalgae removed 73.81–99.14% of DiBP, with *Scenedesmus obliquus* showing the strongest removal. As autotrophic microorganisms, microalgae have been less studied for the degradation of PAEs. Research has primarily focused on their significant functions in aquatic ecosystems. One study reported that *Peniophora lycii* LE-BIN 2142 had the highest degradation efficiency of DEHP [183]. Degradation of PAEs using *Penicillium* sp. resulted in 99.88% removal of DBP within 12 h [178]. Li [184] isolated two *Trichoderma* sp. strains from rhizosphere soils of a moderately contaminated tea plantation; these two *Trichoderma* strains maintained good PAE degradation abilities at different starting concentration. Although fewer studies on PAE degradation by fungi exist, fungal degradation is still a more promising method owing to the special enzymatic repertoires. However, microbial remediation faces limitations owing to numerous influencing factors, which hinder its efficiency and practical application. Therefore, further research is required to optimize its practical application.

### 5.3. Innovative Prevention and Control Technologies

With increasing environmental protection requirements and scientific advancements, PAE prevention and control strategies are gradually shifting from traditional approaches to more efficient, eco-friendly degradation technologies and innovative materials.

Microbial remediation is a promising technology for PAE contamination risk control in crop–soil systems owing to its high environmental compatibility and efficiency [172]. However, the microbial degradation of PAEs mentioned above focuses on the degradation capability of individual bacterial strain and faces significant limitations when applied to complex soil environments. To improve the microbial degradation capacity, degradation stability, and practical applicability in crop–soil systems, researchers have developed a series of novel microbial remediation technologies in recent years, including enzyme degradation, microbial community associations, genetic engineering of microorganisms, immobilized microorganisms, and comprehensive analysis of microorganisms by histology.

Enzymatic degradation has received much attention owing to its high substrate specificity and catalytic efficacy in the elimination of PAEs. Only a limited number of PAE hydrolases have been fully characterized. For example, a hydrolase isolated from *Glutamicibacter* sp. strain 0426 completely degraded DBP within 12 h at 32 °C and pH 6.9. In addition, six novel PAE-degrading enzymes were identified through metagenomics, with esterases exhibiting high hydrolytic activity against DBP at 40 °C and pH 7.5. In agricultural soils, esterase from *Geobacillus* sp. degraded DBP by 59%, while *Bacillus subtilis* esterase degraded DEHP by 35% [185]. Although classical directed evolution strategies have limitations in enhancing the catalytic performance of PAE-degrading enzymes, the integration of big data and artificial intelligence offers new opportunities. Biotechnology, such as enzyme immobilization, whole-cell biocatalysis, and surface display, can be used to further improve the activity and stability of the enzymes. Dong et al. [186] developed a two-step method for magnetic curli nanomaterial preparation to achieve the selective and oriented immobilization of esterases. In another study, esterase from *Bacillus subtilis* was immobilized on halloysite nanotubes (HNTs), which enhanced thermal and storage stability. The resulting HNT–enzyme composite retained strong catalytic activity even after it was reused over seven cycles [187].

The synergistic effects of microbial communities can improve the overall degradation efficiency. Zhang et al. [13] enriched and domesticated a highly efficient and broad-spectrum PAE-degrading endophytic bacterial consortium from PAE-contaminated vegetables, which not only colonized the plants and effectively promoted PAE depuration but also promoted plant growth. In one study, key strains with strong individual degradation ability or beneficial metabolic interactions were co-cultured from maize rhizosphere isolates to form a synthetic bacterial consortium (SC-5). This consortium effectively degraded DBP and DEHP, with removal rates as high as 98.1–100%. Moreover, SC-5 successfully recolonized the maize rhizosphere and significantly enhanced DEHP removal [188]. Zuo et al. [16] applied an endophytic bacterial consortium EN to lettuce through a combination of sprinkler irrigation and drip irrigation treatments. Compared with the uninoculated treatment, this approach significantly improved lettuce growth and reduced PAE residues by 94.05%. The synthetic bacterial colony was effective in alleviating biotic (*Fusarium wilt*) and abiotic (PAEs) stresses in cucumber, and there was a metabolic reciprocity among the strains, which would selectively utilize the provided carbon source (some metabolites of PAEs) for growth [189].

Genetic modification of microorganisms has made it possible to produce more efficient PAE-degrading strains. For example, recombinant *Escherichia coli* expressing esterase genes showed a higher ability to degrade PAEs. In one study, *Pseudomonas* sp. JY-Q was used as a chassis strain, and a JY-Q-R1-R4-SFM-TPH strain was obtained through synthetic biology, which can degrade both PET and PAEs [14]. Xie et al. [190] transformed *Pseudochrobactrum* sp. XF203 into a PAE-degrading strain through gene-directed ribosome engineering to obtain an ideal mutant strain XF203R with high DBP-degrading ability, which can remove DBP from soil by recombinant bacterial communities, showing practical application in DBP bioremediation.

Immobilized microorganisms can exhibit higher stability and activity in the soil environment and provide sustained remediation. In one study, cedar biochar was used as a carrier for the preparation of immobilized bacterial agents (IBAs), which demonstrated efficient and broad-spectrum degradation [15]. Biochar can be used not only as an immobilization carrier but also as a biostimulant. For example, *Arthrobacter* sp. JQ-1 immobilized on maize stover biochar simultaneously adsorbed and degraded DEHP, enhancing microbial abundance, metabolism, and DEHP utilization and providing an effective strategy for the co-remediation of PAE pollution [191]. Tang et al. [192] developed an innovative bifunctional enzyme@MOFs biocomposite with synergistic adsorption and biodegradation functions, which could adsorb 88.56% of DBP in 5 min and degrade 94.6% of DBP in 48 h in an aqueous environment for efficient removal of DBP.

Comprehensive analysis of omics provides insights into microbial communities and their functional genes, enabling the identification of key degradation products and optimization of remediation strategies [193]. Du et al. [194] identified 66 key genes involved in the biodegradation of DBP from *Pseudomonas aeruginosa* PS1 through genomics and transcriptomics analyses. These findings revealed the genetic basis for the bacterium’s catabolic pathway from DBP to succinyl-CoA or acetyl-CoA. The key genera for PAE degradation in purple agricultural soils were screened by metagenomic techniques. These genera promote PAE degradation and maintain the stability of community structure through functions such as ester bond hydrolysis and community sensing [195].

In addition to microbial remediation, novel bio-organic fertilizers can significantly reduce the concentration of PAEs in soil, with a maximum reduction of up to 99.39% [196]. DEHP removal rate in soil treated with Fe_3_O_4_ nanoparticles and compost reached 89.57%, owing to the enhancement of native soil microflora activity by both the compost nutrients and the Fe-based nanoparticle micronutrients [197]. The synergistic ability of Fe_3_O_4_ nanoparticles and compost in assisted bioremediation strategies is considered a promising and environmentally friendly bioremediation strategy in agriculture.

The development of novel treatment technologies and materials provides multiple pathways for PAE removal. Molecularly imprinted polymers (MIPs) are a kind of material with the specific recognition ability of “antibody–antigen,” which utilizes covalent or non-covalent interaction for the pre-assembling of target molecules with the functional monomers, followed by copolymerization with cross-linking agents. Huang et al. [198] constructed a molecularly imprinted electrochemical sensor for the rapid detection of DINP based on the DINP-MIP obtained from the screening for rapid and efficient detection of DINP in edible oil. Wei et al. [199] used *Arundo donax* solid-phase denitrification reactor for simultaneous removal of DBP and nitrogen, and DBP did not affect the activity of denitrifying genera. Sun et al. [200] synthesized a microporous polymer with an adsorption capacity of 787 mg/g of DBP, which reached adsorption equilibrium rapidly, but the presence of ethanol inhibited the polymer performance. Zhao et al. [201] co-immobilized PtCo nano-enzymes with laccase, which acted synergistically with the enzyme via free radicals, and within 72 h, the PAE degradation rate reached 81.83%, and the efficiency remained at 72.44% after five cycles. Furthermore, chitin-based sponges doped with oxygen-modified graphitic carbon nitride (O-gCN) exhibited synergistic adsorption–photocatalytic activity to effectively remove DEP and DBP. The enhanced catalytic activity of O-gCN enhanced the adsorption and photocatalytic effect of ChCN sponges, significantly improving the removal rate of PAEs, with 100% removal of DEP and DBP within 2 h [202].

Therefore, in practical applications, agronomic control measures are more widely adopted, including optimization of irrigation and temperature regimes, application of soil amendments, selection of low-accumulation crop varieties, implementation of intercropping systems, and use of immobilized microbial agents. These approaches have been validated under field conditions and can synergistically achieve the dual objectives of pollution mitigation and sustained agricultural production. The innovative prevention and control measures frequently integrate cutting-edge multidisciplinary technologies, such as biotechnology and environmental engineering, thereby driving progress in agricultural pollution prevention and control. This technological convergence offers enhanced potential for addressing complex environmental challenges.

## 6. Conclusions and Perspectives

The extensive contamination of cash crops with PAEs has attracted widespread attention. It not only affects the commercial value of crops but, more importantly, it can enter the human body via the food chains through bioconcentration in edible parts, posing a potential threat to the health of consumers. Existing studies have shown that PAEs can enter plants in both gaseous and particulate forms through root uptake and foliar adsorption, thus interfering with the normal metabolic processes of plants and ultimately affecting their growth, development, and quality formation. Therefore, developing an efficient, economical, and practical system to prevent and control PAE pollution is of both theoretical and practical importance. This review systematically outlines the pollution characteristics, source analysis, stress effects, and control strategies of PAEs in cash crops, aiming to provide a scientific basis for the safe production of agricultural products and the management of pollutants. The following seven main recommendations are proposed for future research on PAEs in cash crops:

(1) Cash crops play a vital role in dietary structures, with significantly varying capacities for PAE accumulation among different crops. Identifying high-risk crops, such as fruits and roots vegetables, which pose a higher risk to children’s health [5] is essential and requires the development of targeted regulatory measures. In addition, the main sources of PAE contaminants in cash crops are soil, moisture, atmosphere, and packaging materials; however, the relative contributions of these sources have not been evaluated in detail. Therefore, there is an urgent need to strengthen the analysis of PAE enrichment characteristics and pollution sources in cash crops in the future, especially with regard to high-risk substances such as DEHP and DBP. Future research should focus on quantitatively assessing the distribution patterns and environmental contributions of PAEs to enable more effective source control.
(2) Cash crops may also contain different concentrations and forms of other pollutants, such as heavy metals, pesticides, polycyclic aromatic hydrocarbons, and microplastics. Owing to a lack of systematic and comprehensive data support, accurate elucidation of the combined effects of multiple co-existing pollutants, understanding of their mechanisms of action, and assessment of their overall toxicity remain challenging. Therefore, future research should explore the combined toxicity of co-existing pollutants and develop effective removal strategies.
(3) Microbial communities are essential for the effective biodegradation of PAEs. Advances in omics techniques and genetic engineering have provided promising avenues for the identification and enhancement of microbial strains capable of effectively degrading PAEs under different environmental conditions. Chen et al. [203] first reported a synchronous biodegradation system of polycyclic aromatic hydrocarbons (PAHs) and PAEs, which revealed that the bacterial community was enriched by functional genera, regulated by extracellular polymeric substance (EPS) components, and reconfigured via a metabolic network. Therefore, based on the understanding of different single strains, the metabolic interaction network of the bacterial community should be further resolved; the microbial community should be synthesized through genetic engineering, and its long-term stability should be assessed in practical engineering to explore the mechanism of action and bioremediation capability. In addition, exploring the synergistic potential of microbial communities and immobilization techniques could further optimize the biodegradation process. Future research could prioritize the development of more effective, scalable, and environmentally friendly remediation technologies, particularly microbial and bioengineering approaches.
(4) Current microbial remediation studies of PAEs are mainly limited to laboratory conditions, and the practical application of these microorganisms in the environment remains uncertain [17]. The ability to utilize laboratory-screened microorganisms for degradation in real soil environments is a major challenge. Therefore, future efforts should focus on enhancing the applicability of microorganisms under field conditions, overcoming the challenges of limiting factors under field conditions, and developing low-cost remediation methods that are suitable for large-scale use. Such advances will help to effectively reduce or eliminate the impact of PAEs on cash crops.
(5) Although some studies have been conducted on the regulatory effects of agronomic measures on PAEs in crops, the regulatory mechanism remains insufficiently understood. Moreover, there is a lack of practical and regulatory parameters, such as application rates, timing, or environmental thresholds, for implementation in actual farmland conditions. Therefore, it is necessary to conduct in-depth analyses of the physicochemical and microbiological regulatory mechanisms of agronomic measures on the degradation of PAEs in cash crops. This knowledge should inform the development of an integrated regulatory technology of agronomic measures aimed at enhancing PAE degradation in cash crops.
(6) To overcome the limitations of single technologies and improve the removal efficiency of PAEs, the focus of research on PAE prevention and control strategies has shifted in recent years to a synergistic management system that couples multiple treatment technologies. Future research could focus on optimizing the process parameters of the coupled technologies, evaluating their long-term ecological safety, and developing low-cost and scalable engineering applications to facilitate the transformation of PAE pollution prevention and control from laboratory research to actual production.
(7) Encouraging interdisciplinary cooperation is essential for addressing the challenges posed by PAEs, and expertise in agronomy, environmental science, medicine, toxicology, and public health can be integrated under the One Health approach [204]. The complexity of the pollutant management of PAEs requires not only multisectoral and cross-sectoral collaboration among relevant governmental departments but also strengthened cooperation among the international community to formulate global environmental policies and regulations. Such efforts are crucial for promoting source-level prevention and reducing the production and emission of PAEs into the environment.

## Figures and Tables

**Table 1:** Distribution characteristics and risk assessment of PAE contaminants in cash crops. The hazard quotient (HQ) was calculated using the formula HQ = EDI/RfD. The estimated daily intake (EDI) was derived from the measured concentration and typical consumption rates, while the reference dose (RfD) was sourced from the Integrated Risk Information System (IRIS) of the U.S. Environmental Protection Agency. HI: hazard index; CR: cancer risk; SML: specific migration limits; MRL: maximum residue limit; ERL: environment risk limits; R. Detailed information is shown in the Supplementary Materials. LOD: limit of detection; LOQ: limit of quantification; QuEChERS: quick, easy, cheap, effective, rugged, safe; GC-MS/MS: gas chromatograph equipped with a triple quadrupole mass spectrometer; SPE: solid-phase extraction; GC-MS: gas chromatography-mass spectrometry; DLLME: dispersion liquid–liquid microextraction; GC-FID: gas chromatography-flame ionization detector; LLE: liquid-liquid extraction; LC-MS/MS: liquid chromatography-tandem mass spectrometry; MSPE: magnetic solid phase extraction; ILCR: incremental lifetime cancer risk; dw: dry weight; UHPLC-HESI-MS/MS: ultra high-performance liquid chromatography heated electro spray ionisation high resolution mass spectrometry (Orbitrap); LP GC-QqQMS: low-pressure gas chromatography-tandem mass spectrometry; UE: ultrasonic extraction; GC-ECD: gas chromatography-electron capture detector; ND: not detected.

Whether to Process	Variety	Location	N	Extraction Method	Detection Method	LOD (μg/kg)	LOQ (μg/kg)	PAEs (mg/kg)	Risk Assessment	References
processed	tea (packaged)	China	204	QuEChERS	GC-MS/MS	-	-	0.31–8.15	below the safety limit (HQ)	[22]
	tea	Zhejiang, China	6	SPE	GC-MS	0.21–0.61	0.71–2.03	0.46–1.51	below the safety limit (HQ)	[23,24]
	coffee hot drink	Italy	3	SPE, DLLME	GC-FID	0.1–2.9 (μg/mL)	1.2–8.2 (μg/mL)	-	-	[25]
	coffee brew sample	Italy	9	LLE	GC-MS	-	-	159–5305 (μg/L)	-	[26]
	coffee	Turkey	40	QuEChERS	LC-MS/MS	1.18–2.79 (ng/mL)	3.92–9.30 (ng/mL)	3.61–115.20 (ng/mL)	below the safety limit (HQ, HI, CR)	[27]
	orange juice	Tehran, Iran	12	MSPE	GC-MS	0.12–1.26 (μg/L)	0.36–3.78 (μg/L)	3.56–4.4 (μg/L)	below the safety limit (HI, ILCR)	[28]
	apple juice	Tehran, Iran	12	MSPE	GC-MS	0.12–1.26 (μg/L)	0.36–3.78 (μg/L)	3.06–3.79 (μg/L)	below the safety limit (HI, ILCR)	[28]
	peach juice	Tehran, Iran	12	MSPE	GC-MS	0.12–1.26 (μg/L)	0.36–3.78 (μg/L)	3.07–3.46 (μg/L)	below the safety limit (HI, ILCR)	[28]
	pineapple juice	Tehran, Iran	12	MSPE	GC-MS	0.12–1.26 (μg/L)	0.36–3.78 (μg/L)	3.8–5.35 (μg/L)	below the safety limit (HI, ILCR)	[28]
	mango juice	Tehran, Iran	12	MSPE	GC-MS	0.12–1.26 (μg/L)	0.36–3.78 (μg/L)	2.66–2.95 (μg/L)	below the safety limit (HI, ILCR)	[28]
	carrot/dw	Shandong, China; Jiangsu, China; Zhejiang, China;	30	QuEChERS	GC-MS/MS	0.015–0.041	-	6.77–21.54	below the safety limit (HI)	[19]
	cabbage/dw	Shandong, China; Jiangsu, China; Zhejiang, China;	30	QuEChERS	GC-MS/MS	0.015–0.041	-	3.58–37.56	below the safety limit (HI)	[19]
	bean/dw	Shandong, China; Jiangsu, China; Zhejiang, China	30	QuEChERS	GC-MS/MS	0.015–0.041	-	4.88–20.42	below the safety limit (HI)	[19]
	peanut oil (skinned)	Shandong, China	3	SPE	GC-MS	0.05–1.83	0.15–4.10	3.75–4.60	-	[29]
	peanut oil (de-skinned)	Shandong, China	3	SPE	GC-MS	0.05–1.83	0.15–4.10	2.97–3.64	-	[29]
	olive oil	Europe	30	LLE	UHPLC-HESI-MS/MS	20–350	70–1170	6.2–400.5	below the safety limit (SML)	[30]
	olive oil	Italy	23	Dilute-and-Inject	LP GC-QqQMS	4–341	13–1136	ND–66.75	-	[31]
	olive oil	Spain	36	LLE	GC-MS	-	-	ND–3.49	-	[32]
	walnut oil	Xinjiang, China	36	SPE	GC-FID	70–230	220–780	1.96–19.81	below the safety limit (MRL)	[33,34]
	rapeseed oil	Sichuan, China	33	SPE	GC-MS	8–20	6–30	ND–5.69	DEHP,DBP (MRL) ≥ 0.3	[33,35]
	tobacco	Iran; Guizhou, China	200	SPE	GC-MS	0.001–0.003	0.003–0.008	0.001–0.018	below the safety limit (ERL)	[36,37]
raw	tea (fresh)	Zhejiang, China	15	SPE	GC-MS	0.2–0.4	0.67–1.32	0.08–1.91	below the safety limit (HQ)	[24,38]
	apple	Beijing, China; Zhejiang, China	70	QuEChERS, UE	GC-MS GC-MS/MS	1.6–3.7	3.2–7.4	0.08–0.37	below the safety limit (SML)	[39,40]
	avocado	Beijing, China	1	UE	GC-MS/MS	-	-	0.41–0.91	below the safety limit (SML)	[40]
	grape	Xinjiang, China	69	SPE	GC-MS	-	-	0.04–0.13	below the safety limit (HI)	[41]
	peach	Zhejiang, China; Shenyang, China	45	QuEChERS, LLE	GC-MS	0.91–66.97	2.70–200.90	0.05–0.19	below the safety limit (HQ, CR)	[39,42]
	kiwi fruit	Zhejiang, China	40	QuEChERS	GC-MS	1.6–3.7	3.2–7.4	0.08–0.28	-	[39]
	banana	Zhejiang, China	40	QuEChERS	GC-MS	1.6–3.7	3.2–7.4	0.07–0.26	-	[39]
	dragon pearl fruit	Zhejiang, China	40	QuEChERS	GC-MS	1.6–3.7	3.2–7.4	0.08–0.18	-	[39]
	plum	Zhejiang, China	40	QuEChERS	GC-MS	1.6–3.7	3.2–7.4	0.09–0.19	-	[39]
	watermelon	Zhejiang, China	40	QuEChERS	GC-MS	1.6–3.7	3.2–7.4	0.05–0.15	-	[39]
	mango	Zhejiang, China	40	QuEChERS	GC-MS	1.6–3.7	3.2–7.4	0.08–0.19	-	[39]
	orange	Zhejiang, China	40	QuEChERS	GC-MS	1.6–3.7	3.2–7.4	0.05–0.15	-	[39]
	strawberry	Zhejiang, China	40	QuEChERS	GC-MS	1.6–3.7	3.2–7.4	0.06–0.12	-	[39]
	lettuce	California, USA	3	SPE	GC-MS	0.1–26	0.3–72	0.78–1.25	-	[43]
	carrot	California, USA	3	SPE	GC-MS	0.1–26	0.3–72	1.75–3.76	-	[43]
	garlic	Jiangsu, China	11	UE	GC-MS	0.1–0.9	0.5–7.4	4.27–10.13	-	[44]
	celery	Hebei, China; Shandong, China	38	QuEChERS	GC-MS/MS	0.1–1.1 (μg/L)	0.2–3.7 (μg/L)	0.09–2.73	below the safety limit (HI)	[45]
	peanut	Shandong, China	2	SPE	GC-MS	0.05–1.83	0.15–4.10	2.79–3.42	-	[29]
	rapeseed	Sichuan, China	5	SPE	GC-MS	-	-	ND–0.089	-	[35]
	walnut	Xinjiang, China	4	SPE	GC-MS	7–230	220–780	2.51–11.29	below the safety limit (MRL)	[34]
	ginseng	Jilin, China	12	LLE	GC-MS	0.002 (mg/L)	0.010 (mg/L)	0.18–0.55	below the safety limit (HQ)	[46]
	cotton	Xinjiang, China	3	UE	GC-ECD	-	-	41.98–158.87	-	[47]

## Data Availability

No new data were created or analyzed in this study. Data sharing is not applicable to this article.

## Supplementary Materials

The following supporting information can be downloaded at https://www.mdpi.com/article/10.3390/plants15040549/s1, Figure S1. The workflow of creating a VOSviewer network contribution map; Table S1. The top 10 high-frequency keywords in the keyword co-occurrence network; Table S2. The top 10 countries/regions by frequency in the country collaboration network.
