# Supplementary material for: Occurrence, Sources, Phytotoxicity, and Prevention and Control System of Phthalate Esters in Cash Crops: A Comprehensive Review
Source: Plants (Basel). 2026 Feb 10;15(4):549. doi: 10.3390/plants15040549 (PMC12943975; doi:10.3390/plants15040549)
Supplement: Supplementary file 1 [file plants-15-00549-s001.zip › plants-4090819-supplementary.pdf]

Supplementary Materials for

Occurrence, Sources, Phytotoxicity and Prevention and Control System of Phthalate Esters in Cash Crops: A Comprehensive Review

Specific steps for creating a VOSviewer network contribution map

This study employed VOSviewer software to conduct a systematic bibliometric analysis of research literature focusing on "plants" and "phthalates." The specific operational procedure was as follows: First, relevant literature was retrieved and exported in plain text format from the Web of Science Core Collection using a combination of topic-related keywords. In VOSviewer, we initially performed keyword co-occurrence analysis to explore the knowledge structure of the field, with parameters set to the full counting method and a minimum keyword occurrence threshold of 9 for inclusion. Subsequently, country collaboration network analysis was conducted to reveal international cooperation patterns, with the parameter set to a minimum publication count of 5 per country (Cui et al., 2025; Li et al., 2023). Both analyses employed the default resolution (1.0) for modularity clustering to automatically identify research topic communities and national collaboration clusters. Based on this, we generated keyword co-occurrence network maps and country collaboration network maps.

Figure S1 The workflow of creating a VOSviewer network contribution map

Table S1 The top 10 high-frequency keywords in the keyword co-occurrence network

Table S2 The top 10 countries/regions by frequency in the country collaboration network

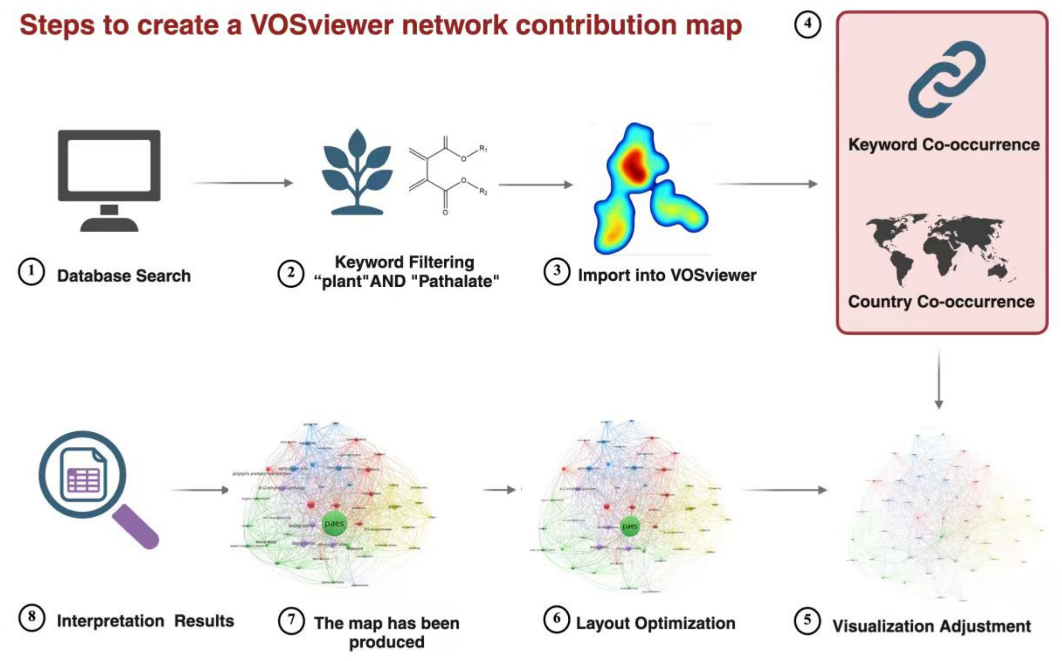

Figure S1 The workflow of creating a VOSviewer network contribution map

Table S1 The top 10 high-frequency keywords in the keyword co-occurrence network

Rank	Keyword	Occurrence	Total Link Strength	Cluste (Color)
1	paes	128	615	1 green
2	degradation	39	213	4 purple
3	contamination	30	199	2 blue
4	soil	38	198	3 red
5	agricultural soils	32	188	2 blue
6	di-(2-ethylhexy)phthalate	32	183	4 purple
7	accumulation	27	169	5 yellow
gu8	growth	31	157	5 yellow
9	biodegradation	28	153	4 purple
10	metabolism	25	151	3 red

Table S2 The top 10 countries by frequency in the country collaboration network

Rank	Country	Occurrence	Total Link Strength	Cluster (Color)
1	China	181	32	2 green
2	USA	17	14	2 green
3	Germany	8	13	3 blue
4	Saudi Arabia	9	9	1 red
5	Australia	5	8	1 red
6	Egypt	6	8	1 red
7	England	5	8	1 red
8	Ireland	5	5	2 green
9	Iran	6	3	3 blue
10	Pakistan	5	2	3 blue

Risk assessment method

"Below the safety limit" indicates that the concentration of the chemical substance in the sample is lower than the maximum residue limit specified in the relevant regulations. "Non-carcinogenic risk" refers to the risk assessment results based on the hazard quotient (HQ) method. If the Hazard Index (HI) is less than 1, it indicates that the cumulative non-carcinogenic risk of multiple chemical substances is within an acceptable range; if the Hazard Quotient (HQ) is less than 1, it indicates that the non-carcinogenic risk of a single chemical substance is within an acceptable range. The Tolerable Daily Intake (TDI) is an estimate of the daily intake of a chemical substance over a lifetime that is not expected to cause observable adverse health effects; for example, the TDI limit for this substance is ≤ 50 μg/kg body weight. The Maximum Residue Limit (MRL) is the highest legally permitted concentration of a chemical residue in food. According to EU regulations, the MRLs for the plasticizers DBP, BBP, and DEHP in specific foods are < 0.3, 30, and 1.5 mg/kg, respectively. The Specific Migration Limit (SML) is the maximum permitted amount of a chemical substance migrating from food contact materials into food. According to Chinese regulations, the SMLs for DBP and DEHP are both ≤ 0.3 mg/kg. The Effects Range-Low (ERL) is a screening benchmark for ecological risk assessment in sediments. When the chemical concentration is below the ERL, it indicates a low ecological risk. In the table, the ERLs for DBP and DNBP are ≤ 0.7 and ≤ 1.0 mg/kg, respectively.

The specific formulas involved are as follows.

$$HQ = E / RfD$$

where HQ is the Hazard Quotient, E is the estimated daily intake, and RfD is the reference dose of the substance. When HQ < 1, the risk is acceptable.

$$HI = \Sigma HQ$$

where HI is the Hazard Index and ΣHQ is the sum of the hazard quotients of all relevant chemical substances. When HI < 1, the cumulative risk is acceptable.

$$C\_sample \leq Limit$$

where C_sample is the measured concentration of the chemical substance in the sample, and Limit refers to the corresponding legal limit (including MRL, SML, etc.).

$$E\_actual \leq TDI$$

where E_actual is the estimated actual daily intake of the population, and TDI is the Tolerable Daily Intake.

$$C\_sediment \leq ERL$$

where C_sediment is the concentration of the chemical substance in the sediment, and ERL is the Effects Range-Low.

References

Cui ZG, Shi C, Zha LT, Liu JM, Guo YC, Li XH, Zhang EJ, Yin ZH. Phthalates in the environment of China: A scoping review of distribution, anthropogenic impact, and degradation based on meta-analysis. *Ecotox. Environ. Safe.* **2025,** 289, No. 117659

Li XX, Wang Q, Jiang N, Lv HJ, Liang CL, Yang HY, Yao XF, Wang J. Occurrence, source, ecological risk, and mitigation of phthalates (PAEs) in agricultural soils and the environment: A review, *Environ. Res.* **2023,** 220, No.115196.
